# Astragaloside IV attenuates oxidative stress-associated mitophagy-dependent ferroptosis in peritoneal mesothelial cells via suppression of STAT3 signaling

**DOI:** 10.3389/fphar.2026.1747615

**Published:** 2026-07-30

**Authors:** Sijia Liu, Li Sheng, Xiaoqian Chen, Jinyi Sun, Xiaohui Meng, Yongqing You, Funing Wang, Manshu Yu, Yun Shan, Meixiao Sheng

**Affiliations:** 1 Department of Nephrology, Affiliated Hospital of Nanjing University of Chinese Medicine, Nanjing, China; 2 Medical Experimental Research Center, First College of Clinical Medicine, Nanjing University of Chinese Medicine, Nanjing, China; 3 First College of Clinical Medicine, Nanjing University of Chinese Medicine, Nanjing, China

**Keywords:** astragaloside IV, ferroptosis, mitophagy, oxidative stress, peritoneal injury

## Abstract

**Background:**

Peritoneal injury and subsequent fibrosis driven by oxidative stress are major contributors to peritoneal dialysis (PD) withdrawal. Ferroptosis and mitophagy are closely correlated with oxidative stress, but their roles and interplay in PD-related peritoneal injury remain incompletely defined. Astragaloside IV (AS-IV) has shown anti-fibrotic and antioxidant potential, yet its underlying mechanism in this context is not fully understood. This study aims to investigate the involvement of mitophagy and ferroptosis during PD and to evaluate whether AS-IV attenuates PD-related peritoneal injury by targeting mitophagy-dependent ferroptosis via STAT3 signaling.

**Methods:**

Mouse models of peritoneal fibrosis were established using either methylglyoxal or PD fluid (PDF). Peritoneal tissues were analyzed by histopathology and Western blotting, and serum malondialdehyde was measured. *In vitro*, human peritoneal mesothelial cells (PMCs; HMrSV5) were assigned to different groups and exposed separately to PDF, carbonyl cyanide m-chlorophenylhydrazone, erastin, or colivlin, followed by separate treatment with N-acetylcysteine, mitochondrial division inhibitor 1, S3I-201, or AS-IV. Ferroptosis and mitophagy were assessed by fluorescence staining and Western blotting, and mitochondrial ultrastructure was evaluated by transmission electron microscopy.

**Results:**

PDF exposure induced oxidative stress-associated mitophagy and ferroptosis in PMCs, with evidence supporting a close association between these processes. AS-IV treatment attenuated STAT3 activation, reduced mitophagy-related changes, and alleviated ferroptosis and peritoneal injury.

**Conclusion:**

Oxidative stress-associated, mitophagy-dependent ferroptosis contributes to PD-related peritoneal injury. AS-IV attenuates this process via suppression of STAT3 signaling in PMCs.

## 1 Introduction

Peritoneal dialysis (PD) is an alternative treatment for end-stage renal disease, with approximately 11% of dialysis patients worldwide undergoing PD therapy (Teitelbaum, 2021; Bello et al., 2022). Peritoneal injury and subsequent fibrosis are major contributors to peritoneal dysfunction, limiting the long-term feasibility of PD (Ito et al., 2024). Oxidative stress is a key driver of peritoneal injury, triggered by biologically incompatible peritoneal dialysis fluid (PDF), uremic toxins, and a systemic pro-inflammatory state (Turner et al., 2014; Roumeliotis et al., 2019; Roumeliotis et al., 2020). Oxidative stress regulators, reactive oxygen species (ROS), can modulate various cell signaling pathways, including by promoting NF-κB and signal transducer and activator of transcription 3 (STAT3) phosphorylation. Additionally, ROS are involved in reciprocal feedback loops with growth factors and cytokines, wherein ROS induce or modulate the production of these factors, and the factors in turn regulate ROS levels (Prasad et al., 2017). Previous studies have shown that the STAT3 pathway is activated during peritoneal fibrosis (Sun et al., 2023). Therefore, this study will further investigate the pathological mechanisms caused by oxidative stress-related STAT3 activation, and may provide effective strategies for preventing and treating peritoneal fibrosis.

Ferroptosis is an iron-dependent form of regulated cell death characterized by intracellular iron accumulation and lipid peroxidation (Jiang et al., 2021). It is associated with intracellular ROS and lipid hydroperoxide (LPO) levels, which are key indicators of oxidative stress. Recent studies have demonstrated its role in the progression of pulmonary fibrosis (Pei et al., 2022), liver fibrosis (Luo et al., 2022), and myocardial fibrosis (Zhang et al., 2022). However, evidence for its involvement in peritoneal fibrosis remains limited but emerging. Transferrin has been detected in the dialysate effluent of patients undergoing PD, and acidic peritoneal dialysate rapidly (<1 min) releases iron from transferrin (Aldriwesh et al., 2019). Additionally, intravenous iron sucrose has been shown to induce inflammation and oxidative stress in peritoneal mesothelial cells (PMCs), impairing cell viability (Mitsopoulos et al., 2020). Previous studies have shown that the secretion of monocyte chemoattractant protein-1 by PMCs is increased following incubation with iron sucrose (Jasinski and Breborowicz, 2022). These findings suggest that ferroptosis driven by iron metabolism dysregulation may contribute to peritoneal injury in PMCs.

Mitochondrial homeostasis plays a vital role in cell survival by regulating energy metabolism and redox balance during ferroptosis (Jiang et al., 2025). Elevated intracellular ROS levels can trigger mitochondrial oxidative damage, ultimately leading to mitophagy (Li et al., 2023). Mitophagy, a selective autophagy process that degrades damaged mitochondria, is essential for maintaining mitochondrial homeostasis (Lu et al., 2023). Mitophagy-dependent ferroptosis has been implicated in liver fibrosis (Bi et al., 2024), Alzheimer’s disease (Li J. H. et al., 2022), and Parkinson’s disease (Zhang et al., 2024). As demonstrated in previous studies, mitophagy plays a dual role in ferroptosis. On one hand, protective mitophagy alleviates ferroptosis by reducing mitochondrial ROS (Yamashita et al., 2024). On the other hand, mitophagy activation exacerbates ferroptosis by increasing intracellular free fatty acids or promoting ferritinophagy (Yu et al., 2022; Yang et al., 2023). However, whether mitophagy-dependent ferroptosis contributes to PD-related peritoneal injury has not been investigated.

Astragaloside IV (AS-IV), a main metabolite extracted from the Chinese medicinal botanical drug Astragalus membranaceus, serves as a quality marker for this botanical drug. Previous studies by the authors demonstrated the therapeutic potential of Astragalus membranaceus in peritoneal fibrosis (Huang et al., 2024; Sheng et al., 2024). Recent pharmacological studies have shown that AS-IV has antioxidant activity and can reduce ROS generation to protect the peritoneum during PD. AS-IV has been shown to alleviate peritoneal fibrosis by modulating the TGF-β/Smad signaling pathway (Zhang et al., 2015) and macrophage-derived exosomes (Shan et al., 2024). Despite extensive studies on AS-IV, its effects on mitophagy and ferroptosis in PMCs remain poorly understood. Therefore, this study aimed to identify the pathological mechanisms of oxidative stress in PMCs and the pharmacologic effects of AS-IV on oxidative stress-induced mitophagy-dependent ferroptosis in PMCs.

## 2 Materials and methods

### 2.1 Antibodies and reagents

Antibodies against signal transducer and activator of transcription 3 (STAT3; sc-8019), phosphorylated-STAT3 (sc-8019), and β-actin (sc-47778) were purchased from Santa Cruz Biotechnology (Santa Cruz, CA). Antibodies against prostaglandin-endoperoxide synthase 2 (PTGS2; 66351-1-Ig), glutathione peroxidase 4 (GPX4; 67763-1-Ig), PTEN induced putative kinase 1(PINK1; 23274-1-AP), BCL2 interacting protein 3 (BNIP3; 68091-1-Ig), FUN14 domain-containing protein 1 (FUNDC1; 28519-1-AP), light chain 3 (LC3; 14600-1-AP), p62 (18420-1-AP), fibronectin (FN; 66042-1-Ig), collagen I (14695-1-AP) were purchased from Proteintech (Wuhan, Hubei Province, China). Antibody against 4-Hydroxy-2-nonenal (4-HNE; bs-6313R) was purchased from Bioss (Beijing, China). Astragaloside IV (AS-IV; #84,687–43–4) (purity ≥ 98%) was purchased from Yuanye Bio-Technology (Shanghai, China). The chemicals methylglyoxal (MGO; HY-w020014), S3I-201 (HY-15146), colivlin (HY-P1061), liproxstatin-1 (HY-12726), Carbonyl cyanide m-chlorophenylhydrazone (CCCP; HY-100941), erastin (HY-15763), N-acetylcysteine (NAC; HY-B0215), and mitochondrial division inhibitor 1 (Mdivi-1; HY-15886) were purchased from MedChemExpress (Monmouth Junction, NJ, USA). The dextrose PD fluid (PDF) was purchased from Baxter Healthcare (Guangzhou, Guangdong Province, China). The manufacturers of all other chemicals and reagents will be specified where mentioned.

### 2.2 Animals

C57BL/6J mice (6–8weeks, 20–25g, male) were purchased from Vital River Laboratory Animal Technology Co., Ltd. (Beijing, China). The animal experiment was approved by the Animal Ethics Committee of Nanjing University of Chinese Medicine (Approval number: A240104) and conducted in accordance with the Guide for the Care and Use of Laboratory Animals (National Institutes of Health, USA, 1985). Following a 1-week acclimatization period, mice were randomly divided into two experiments: MGO experiment to investigate oxidative injury in the process of peritoneal fibrosis, and PD experiment to explore the role of STAT3 in peritoneal fibrosis during PD. Mice in MGO experiment were randomly divided into three groups (n = 5): control, MGO, and MGO + AS-IV. Mice in PD experiment were randomly divided into four groups (n = 5): control, PD, PD + AS-IV, and PD + S3I-201 (the STAT3 inhibitor). Mice in the MGO and PD groups were used as disease models and were intraperitoneally injected once daily at a consistent abdominal injection site with either 40 mM MGO in saline or 4.25% dextrose PDF (100 mL/kg). In the treatment groups, mice were administered the corresponding MGO solution or 4.25% dextrose PDF supplemented with AS-IV (15 mg/kg) or S3I-201 (10 mg/kg), whereas control mice received an equivalent volume of saline (Shan et al., 2024; Song Q. H. et al., 2024). After 4 weeks of daily injections, the mice were euthanized under sodium pentobarbital anesthesia for sample collection.

### 2.3 Cell culture

Human peritoneal mesothelial cell line (HMrSV5, CVCL_UZ40; LM8C0882, LAMI-Bio Co., Ltd.) were cultured in RPMI-1640 medium (KGL1501-500, KeyGEN BioTECH Corp., Ltd.) supplemented with 10% fetal bovine serum (KGA6006-10, KeyGEN BioTECH Corp., Ltd.) and 1% penicillin/streptomycin in a 5% CO_2_ atmosphere at 37 °C. The cells were allowed to attach for 24 h and were grown to 80% confluence.

In the model group, cells were treated with 3% dextrose PDF for 24 h. In different experiments, cells were respectively treated with ROS scavenger NAC (5 mM), mitophagy inducer CCCP (10 μM), mitophagy inhibitor Mdivi-1 (10 μM), ferroptosis inducer Erastin (10 μM), ferroptosis inhibitor Liproxstatin-1 (200 nM), STAT3 inhibitor S3I-201 (100 μM), STAT3 inducer Colivlin (1 μM), and AS-IV (200 μM). CCK-8 assays showed that treatment with AS-IV up to 200 μM for 24 h did not induce a significant increase in proliferation or evidence of cytotoxicity or apoptosis (Supplementary Information A).

### 2.4 Histology, immunohistochemical (IHC), and immunofluorescence (IF) analysis

The parietal peritoneal tissue from the side opposite to the injection site was used for histological analysis. The samples (n = 3) were fixed in 4% formaldehyde for 24 h, embedded in paraffin, then sectioned to 6–8 μm-thick for staining. Hematoxylin and eosin staining and Masson’s trichrome staining were performed to assess peritoneal thickness and pathological changes. For IHC and IF analysis, sections were incubated overnight at 4 °C with a primary antibody, followed by corresponding secondary antibodies. In IHC analysis, paraffin sections were deparaffinized, rehydrated, and subjected to heat-mediated antigen retrieval in EDTA buffer (G1207-1L, Servicebio; 1 mM, pH 8.0) using a 98 °C water bath for 20 min. Endogenous peroxidase activity was then blocked by incubation with 3% H_2_O_2_ solution at room temperature for 25 min. After blocking with 3% BSA in TBST for 30 min, sections were incubated with primary antibodies against fibronectin, collagen I, or 4-Hydroxy-2-nonenal, followed by corresponding secondary antibodies. In IF analysis, the samples were incubated with fluorescently labeled secondary antibodies, Goat Anti-Rabbit IgG H&L (Alexa Fluor® 594) (1:1,000, Abcam ab150080) and Goat Anti-Rabbit IgG H&L (Alexa Fluor® 488) (1:1,000, Abcam ab150077), counterstained with 4′,6-diamidino-2-phenylindole (DAPI), mounted on slides, and observed under an inverted fluorescence microscope (SMI3000B, Leica). The sections were processed by a technician blinded to group allocation.

### 2.5 Serum malondialdehyde (MDA) assay

Serum samples were collected from mice to determine MDA concentrations using a lipid oxidation assay kit (S0131S, Beyotime). Samples and standards were added to the plate for incubation. After the substrate solution was added to initiate the colorimetric reaction, absorbance was measured to determine the MDA concentration.

### 2.6 Mitochondrial membrane potential detection

Cells were incubated with JC-1 solution (C2006; Beyotime) at 37 °C in the dark for 20 min. After incubation, the excess solution was removed, and the cells were washed with JC-1 staining buffer before the fresh medium was added. Fluorescence intensity was observed using an inverted fluorescence microscope (SMI3000B, Leica).

### 2.7 Total ROS and mitochondrial ROS (mtROS) detection

Frozen tissue samples were incubated with dihydroethidium (DHE) solution to assess ROS production in the peritoneum. In cultured cells, intracellular total ROS was measured using 2′,7′-dichlorodihydrofluorescein diacetate solution (DCFH-DA; R253, Dojindo), while mtROS was measured using mitochondrial superoxide indicator Deep Red (MitoSOX Deep Red; MT14, Dojindo). Cells were incubated with each probe at 37 °C for 30 min in the dark, washed with PBS, and imaged using an inverted fluorescence microscope (SMI3000B, Leica) under identical acquisition settings. For DCFH-DA and MitoSOX Deep Red, images were acquired at Ex/Em 488/500–550 nm and 561/640–700 nm, respectively. Fluorescence intensity was quantified using ImageJ with whole-field analysis. For each biological replicate, three random non-overlapping fields were analyzed. Background-corrected mean fluorescence intensity was calculated as: corrected fluorescence intensity = integrated density − (area × mean background fluorescence). Data were normalized to the control group and are presented as fold change.

### 2.8 Detection of intracellular Fe^2+^ levels

After three washes with medium, cells were incubated with FerroOrange solution (F374, Dojindo) at 37 °C for 30 min. Fluorescence intensity was then observed using an inverted fluorescence microscopy (SMI3000B, Leica). ImageJ software was used to quantify the fluorescence intensity, with quantification as described above.

### 2.9 Detection of cellular lipid peroxidation levels

Cells were washed and incubated with BODIPY 581/591 C11 solution at 37 °C for 30 min. Then, green and red fluorescence intensities were observed using an inverted fluorescence microscope (SMI3000B, Leica).

### 2.10 Mitochondrial and lysosomal colocalization assay

Cells were incubated with pre-warmed Mito-Tracker Green solution at 37 °C for 30 min. After removing the solution, pre-warmed Lyso-Tracker Red solution was added, and cells were incubated for an additional 60 min at 37 °C. Finally, a pre-warmed medium was added, and green and red fluorescence intensities were observed using an inverted fluorescence microscope (SMI3000B, Leica).

### 2.11 Transmission electron microscopy (TEM)

Cells were harvested, centrifuged, and fixed in 2.5% glutaraldehyde at 4 °C for 2 h, followed by post-fixation, dehydration, and embedding. Ultrathin sections (70 nm) were prepared, stained with 3% uranyl acetate for 8 min and 2.7% lead citrate for 8 min, then washed and imaged using an FEI Tecnai transmission electron microscope. Uranyl acetate staining was used to enhance electron contrast of cellular ultrastructures, facilitating visualization of mitochondrial morphology.

### 2.12 Western blot (WB) analysis

Total protein was extracted from HMrSV5 cells or mouse peritoneal tissue using RIPA buffer (89901, Thermo Scientific) supplemented with a phosphatase and protease inhibitor mixture. Protein concentrations were determined using the Pierce BCA Protein Assay Kit (A65453, Thermo Fisher Scientific). Then, 20 μg of proteins were mixed with an equal volume of 4× Loading buffer, boiled for 5 min, electrophoresed and transferred to PVDF membranes. After blocking with 5% BSA in TBST for 1 h, membranes were incubated overnight at 4 °C with primary antibodies (1:1,000 dilution). After washes with TBST, membranes were incubated with HRP-conjugated secondary rabbit/mouse IgG antibodies (1:10000 dilution; RGAR001/RGAM001, Proteintech) for 1 h at room temperature. Protein bands were detected using the Pierce ECL WB substrate (WP20005, Thermo Fisher Scientific).

### 2.13 Statistical analysis

Data were analyzed using SPSS software (version 12.0; SPSS Inc., Chicago, IL, USA). For *in vitro* experiments, each condition was tested using n = 3 independent biological replicates. For *in vivo* experiments, Western blot analyses were performed with n = 4 mice per group. Sample sizes were selected based on common practice in mechanistic studies and experimental feasibility. One-way ANOVA followed by Tukey’s multiple comparisons test were used to test the differences between the groups. Data are presented as mean ± standard deviation (SD), and a P < 0.05 was considered statistically significant.

## 3 Results

### 3.1 AS-IV attenuates oxidative stress-induced mitochondrial damage, ferroptosis, and STAT3 phosphorylation in PMCs

**FIGURE 1:**
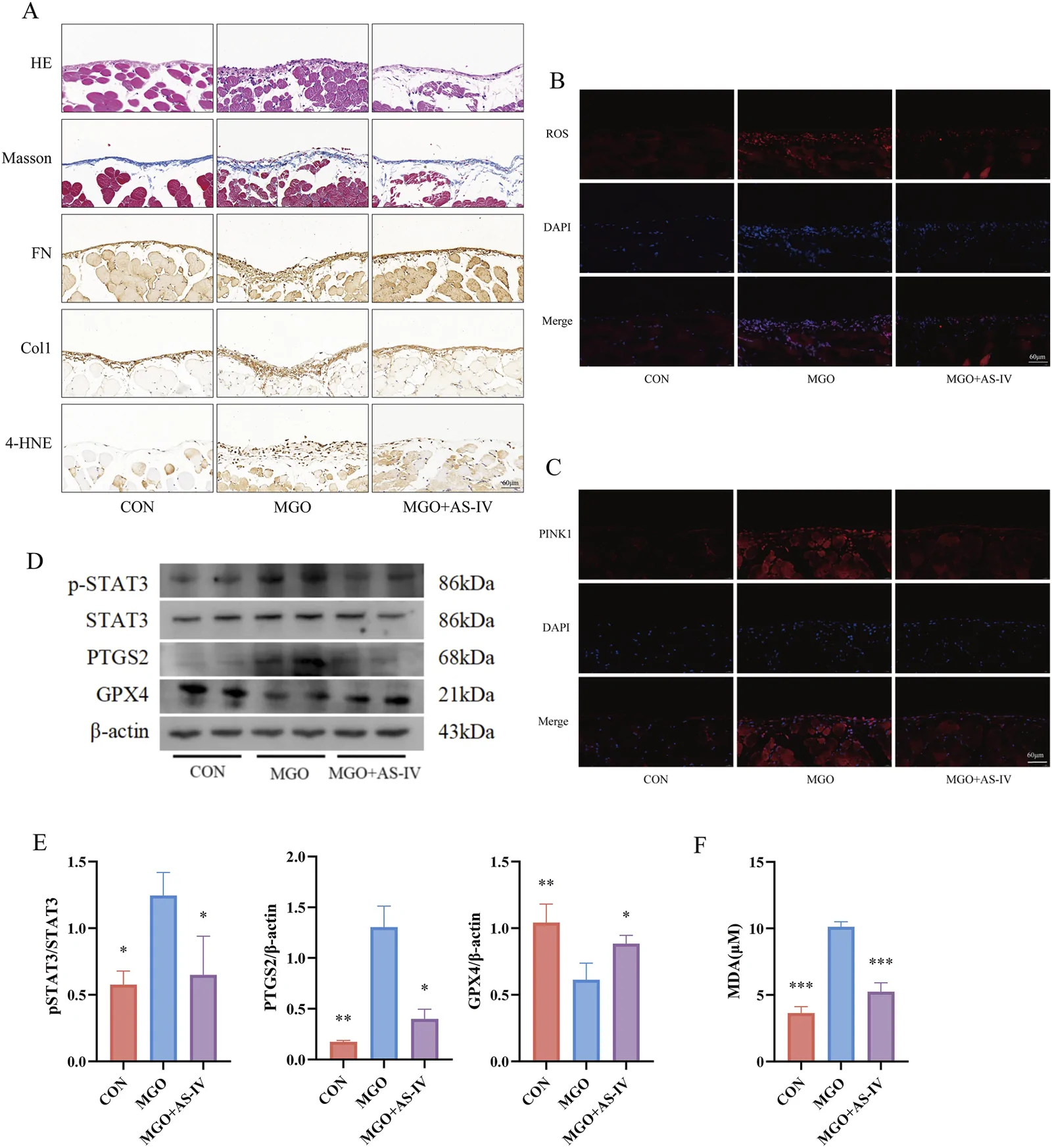
AS-IV mitigates oxidative stress-induced peritoneal injury. C57BL/6 mice were intraperitoneally injected daily for 4 weeks with saline (Control), 40 mM MGO (100 mL/kg, MGO), or 40 mM MGO plus AS-IV (15 mg/kg, MGO + AS-IV). Samples were collected after 4 weeks. **(A)** Peritoneal tissues were subjected to HE staining, Masson staining, and immunohistochemical staining (n = 3). Scale bar, 60 μm. **(B)** ROS levels in peritoneal tissues were detected by DHE fluorescence staining (DAPI, blue; DHE, red) (n = 3). Scale bar, 60 μm. **(C)** PINK1 expression in peritoneal tissues was assessed by immunofluorescence staining (DAPI, blue; PINK1, red) (n = 3). Scale bar, 60 μm. **(D,E)** Expression of p-STAT3, STAT3, PTGS2, and GPX4 in peritoneal tissues was analyzed by Western blotting. Band intensities of PTGS2 and GPX4 were quantified by normalization to β-actin, whlie p-STAT3 was quantified as the p-STAT3/STAT3 ratio (n = 4). **(F)** Serum MDA levels (μM) were measured in each group (n = 3). Data are presented as mean ± SD. One-way ANOVA with Tukey’s multiple-comparisons test was used. *p < 0.05, **p < 0.01, ***p < 0.001 vs. MGO.

To investigate the role of oxidative stress in peritoneal injury, an MGO-induced peritoneal oxidative injury model was established. AS-IV was delivered by intraperitoneal injection to directly act on peritoneal tissues, which may partly avoid the limitation of low oral bioavailability reported for AS-IV (Du et al., 2005; Zhang et al., 2007). MGO-treated mice exhibited significant peritoneal thickening, accompanied by inflammatory infiltration and collagen deposition, as indicated by the upregulation of fibronectin (FN) and collagen 1 expression (Figure 1A). The levels of 4-HNE and MDA is a marker of oxidative stress-induced lipid peroxidation. ROS levels and 4-HNE expression were elevated (Figures 1A,B), and serum MDA levels were significantly increased (Figure 1F), collectively indicating peritoneal oxidative injury.

Next, we explored the effects of oxidative stress on mitophagy and ferroptosis. Immunofluorescence analysis revealed increased PINK1 expression in the peritoneal mesothelium of MGO-treated mice, indicating mitochondrial damage (Figure 1C). Increased phosphorylation of STAT3 was also observed, together with the upregulation of ferroptosis marker PTGS2 and the downregulation of GPX4, suggesting that mitophagy-related signaling was activated and ferroptosis occurred (Figures 1D,E). However, AS-IV treatment reversed these alterations, alleviating peritoneal oxidative damage, suppressing the expression of mitophagy-related markers and STAT3 phosphorylation, and reducing ferroptosis in mice.

### 3.2 Oxidative stress inhibition alleviates PDF-induced mitochondrial damage and ferroptosis in PMCs

**FIGURE 2:**
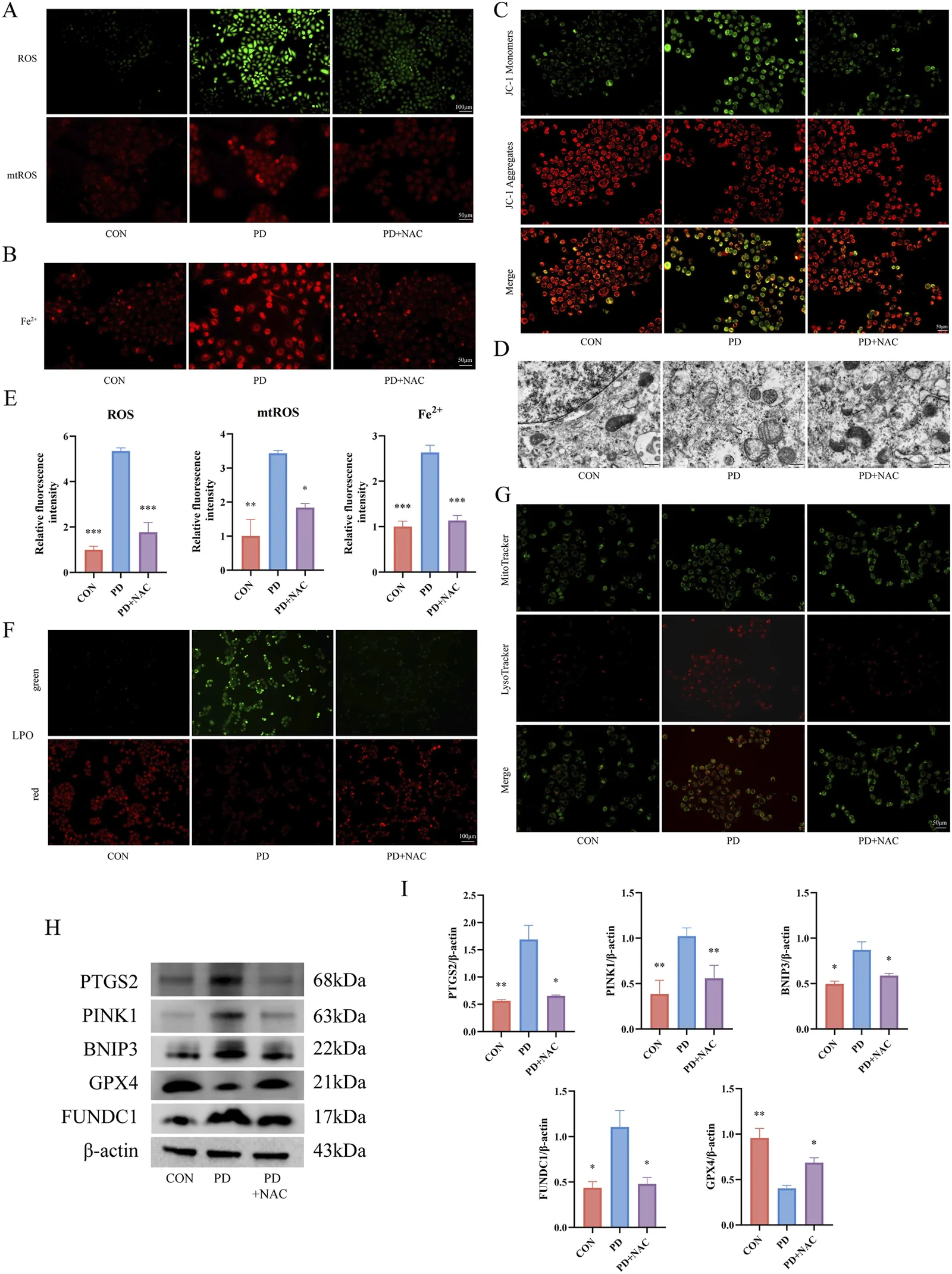
Oxidative stress inhibition attenuates PDF-induced mitochondrial damage and ferroptosis in PMCs. HMrSV5 cells were treated with 3% dextrose PDF in the absence or presence of NAC (5 mM) for 24 h (groups: Control, PD, PD + NAC). **(A–C)** Representative fluorescence images showing intracellular ROS (DCFH-DA), mitochondrial ROS (MitoSOX Deep Red), Fe^2+^ (FerroOrange), and mitochondrial membrane potential (JC-1) in HMrSV5 cells. Scale bars, 50 μm (mtROS and Fe^2+^) and 100 μm (ROS). **(D)** Representative transmission electron microscopy images of HMrSV5 cells. Scale bar, 500 nm. **(E)** Quantitative analysis of ROS, mtROS, and Fe^2+^ levels (n = 3). **(F,G)** Representative fluorescence images showing LPO (C11-BODIPY 581/591) and mitochondria-lysosome colocalization (MitoTracker Green/LysoTracker Red) in HMrSV5 cells. Scale bars, 100 μm **(F)** and 50 μm **(G)**. **(H,I)** Expression of PTGS2, PINK1, BNIP3, GPX4, and FUNDC1 in HMrSV5 cells was analyzed by Western blotting, and band intensities were quantified by normalization to β-actin (n = 3). Data are presented as mean ± SD. One-way ANOVA with Tukey’s multiple-comparisons test was used. *p < 0.05, **p < 0.01, ***p < 0.001 vs. PD.

To examine the impact of oxidative stress on mitochondrial damage and ferroptosis in PMCs, a PDF-induced PMC injury model was established, and the cells were treated with the ROS scavenger NAC. Fluorescent probe staining revealed that PDF exposure increased intracellular ROS and mtROS production (Figures 2A,E). JC-1 staining showed enhanced green fluorescence and diminished red fluorescence in the PD group, indicating a decrease in mitochondrial membrane potential and suggesting mitochondrial dysfunction (Figure 2C). NAC treatment significantly reduced ROS and mtROS levels, inhibiting oxidative stress in PMCs, restoring mitochondrial membrane potential, and alleviating intracellular mitochondrial dysfunction.

TEM showed altered mitochondrial ultrastructure in the PD group, characterized by reduced or absent cristae and vacuolated mitochondria (Figure 2D). Fluorescent probe staining demonstrated increased red fluorescence intensity for Fe^2+^ in the PD group, whereas for LPO, green fluorescence intensity was enhanced and red fluorescence intensity was decreased, indicating elevated intracellular Fe^2+^ and LPO levels. NAC treatment preserved mitochondrial integrity, improved ultrastructural features, and partially alleviated mitochondrial damage, reducing intracellular Fe^2+^ and LPO levels (Figures 2B,E,F). WB analysis further revealed that NAC treatment partially reversed the PDF-induced reduction in GPX4 expression and the increase in PTGS2 expression, suggesting that NAC inhibited PDF-induced oxidative stress and subsequent ferroptosis in PMCs (Figures 2H,I).

Depolarization of the mitochondrial membrane under ROS stimulation triggered mitophagy, leading to the formation of mitochondrial autophagosomes that fuse with lysosomes (Lu et al., 2023). Fluorescence staining showed increased colocalization of mitochondrial green fluorescence and lysosomal red fluorescence in the PD group, indicating enhanced fusion of mitochondria and lysosomes and, thus, that PDF induced mitophagy. NAC treatment significantly reduced mitochondrial damage levels (Figure 2G). WB analysis confirmed that mitophagy-related proteins (PINK1, BNIP3, and FUNDC1) were significantly upregulated in the PD group and reversed by NAC treatment, consistent with the fluorescence staining results (Figures 2H,I).

### 3.3 Mitophagy induction exacerbates ferroptosis in PMCs

**FIGURE 3:**
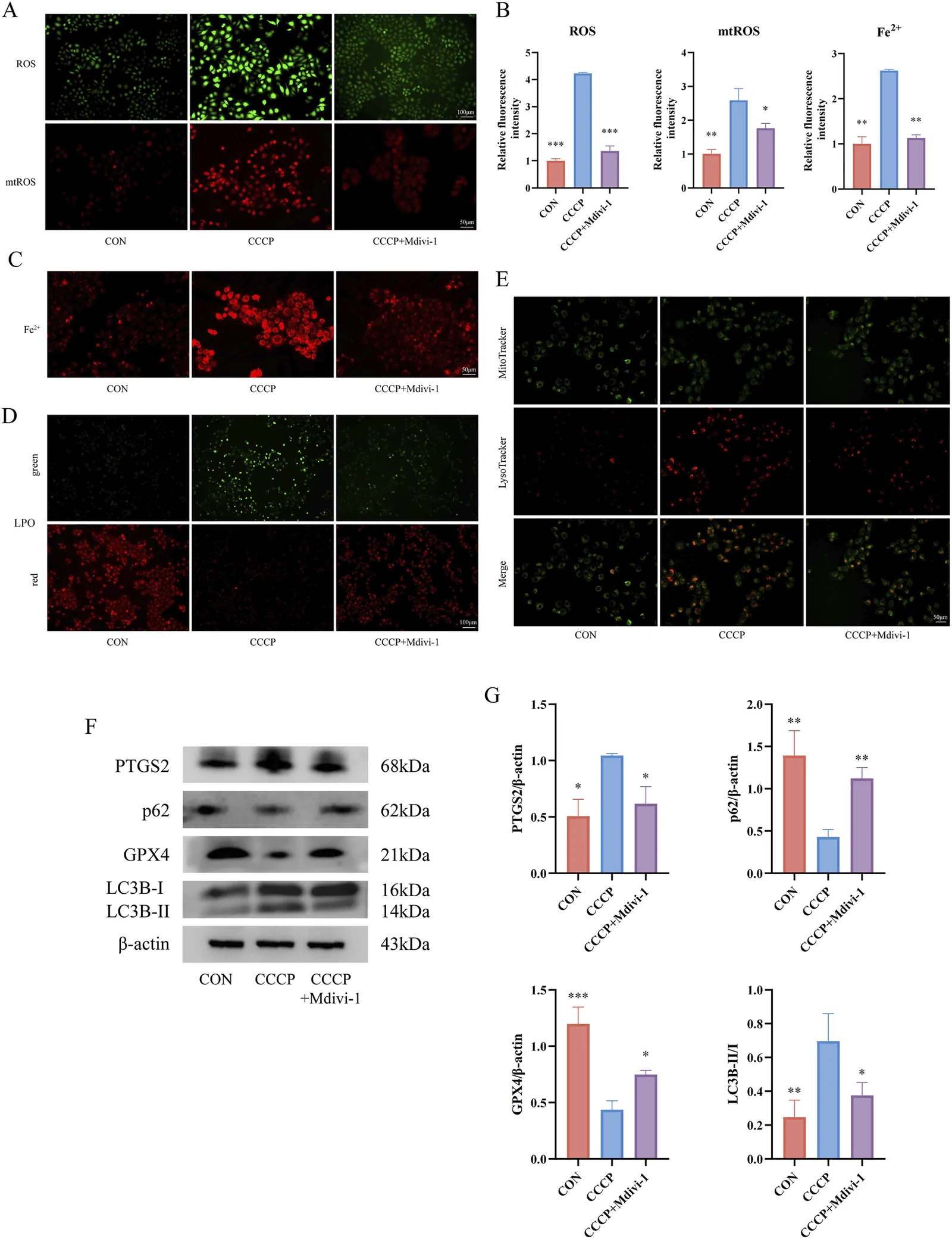
Mitophagy induction exacerbates ferroptosis in PMCs. HMrSV5 cells were treated with CCCP (10 μM) in the absence or presence of Mdivi-1 (10 μM) for 24 h (groups: Control, CCCP, CCCP + Mdivi-1). **(A–E)** Representative fluorescence images showing intracellular ROS (DCFH-DA), mitochondrial ROS (MitoSOX Deep Red), Fe^2+^ (FerroOrange), LPO (C11-BODIPY 581/591), and mitochondria-lysosome colocalization (MitoTracker Green/LysoTracker Red) in HMrSV5 cells. Scale bars, 50 μm (mtROS, Fe^2+^, and colocalization) and 100 μm (ROS and LPO). Quantitative analysis of ROS, mtROS, and Fe^2+^ levels is shown (n = 3). **(F,G)** Expression of PTGS2, p62, GPX4, and LC3B in HMrSV5 cells was analyzed by Western blotting (n = 3). Band intensities of PTGS2, p62, and GPX4 were quantified by normalization to β-actin, while LC3B was quantified as the LC3B-II/LC3B-I ratio (n = 3). Data are presented as mean ± SD. One-way ANOVA with Tukey’s multiple-comparisons test was used. *p < 0.05, **p < 0.01, ***p < 0.001 vs. CCCP.

To further explore the relationship between mitophagy and ferroptosis, we used CCCP, a mitophagy inducer, to analyze whether mitophagy can cause ferroptosis. Treatment with CCCP significantly increased intracellular ROS, mtROS, Fe^2+^, and LPO levels (Figures 3A–D). WB analysis revealed an increased LC3B-II/I ratio and decreased p62 expression, along with decreased GPX4 expression and increased PTGS2 expression, indicating that CCCP induced mitophagy and ferroptosis in PMCs (Figures 3F,G). Compared to the control group, CCCP-treated PMCs exhibited increased fluorescence colocalization of mitochondria and lysosomes, suggesting enhanced mitophagy (Figure 3E). Treatment with the mitophagy inhibitor, Mdivi-1, significantly reduced CCCP-induced mitophagy and ferroptosis, indicating a potential positive feedback mechanism between these two processes.

### 3.4 Inhibition of mitophagy suppresses ferroptosis in PMCs

**FIGURE 4:**
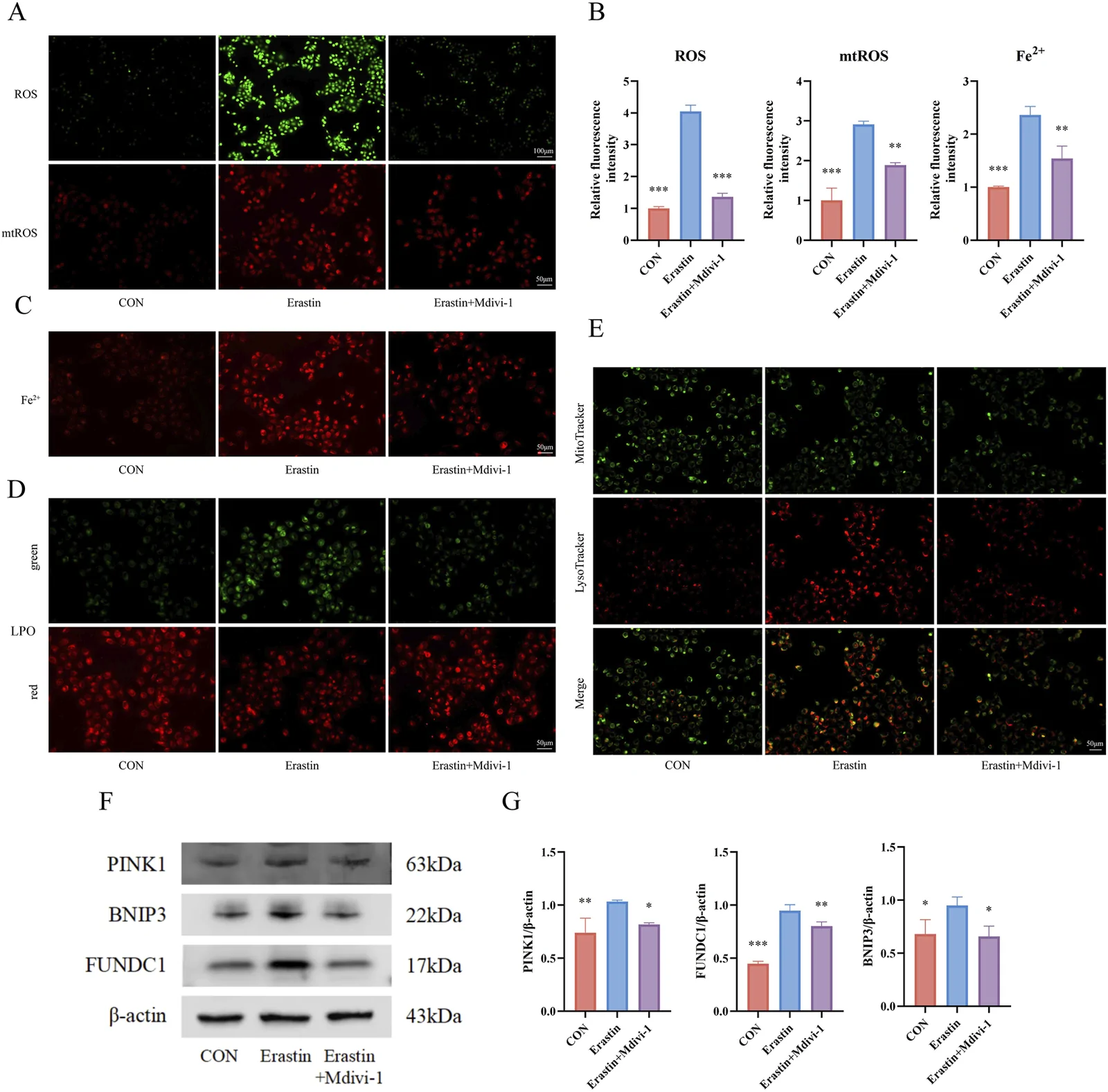
Inhibition of mitophagy suppresses ferroptosis in PMCs. HMrSV5 cells were treated with erastin (10 μM) in the absence or presence of Mdivi-1 (10 μM) for 24 h (groups: Control, Erastin, Erastin + Mdivi-1). **(A–E)** Representative fluorescence images showing intracellular ROS (DCFH-DA), mitochondrial ROS (MitoSOX Deep Red), Fe^2+^ (FerroOrange), LPO (C11-BODIPY 581/591), and mitochondria-lysosome colocalization (MitoTracker Green/LysoTracker Red) in HMrSV5 cells. Scale bars, 50 μm (mtROS, Fe^2+^, LPO, and colocalization) and 100 μm (ROS). Quantitative analysis of ROS, mtROS, and Fe^2+^ levels is shown (n = 3). **(F,G)** Expression of PINK1, BNIP3, and FUNDC1 in HMrSV5 cells was analyzed by Western blotting, and band intensities were quantified by normalization to β-actin (n = 3). Data are presented as mean ± SD. One-way ANOVA with Tukey’s multiple-comparisons test was used. *p < 0.05, **p < 0.01, ***p < 0.001 vs. Erastin.

Erastin treatment was used to further verify the regulation of ferroptosis by mitophagy. Erastin exposure increased intracellular ROS, mtROS, and Fe^2+^ fluorescence intensities while weakening LPO red fluorescence intensity, enhancing green fluorescence, and promoting mitochondrial-lysosomal colocalization (Figures 4A–E). These findings suggest that erastin treatment induces mitophagy and ferroptosis. Treatment with Mdivi-1 partially reduced intracellular ROS, mtROS, Fe^2+^, and LPO levels and decreased mitochondrial-lysosomal colocalization. WB analysis showed that erastin significantly upregulated mitophagy-related proteins (Figures 4F,G), which were reversed by Mdivi-1, suggesting that mitophagy inhibition suppressed erastin-induced ferroptosis.

### 3.5 AS-IV attenuates PDF-induced mitophagy to alleviate ferroptosis in PMCs

**FIGURE 5:**
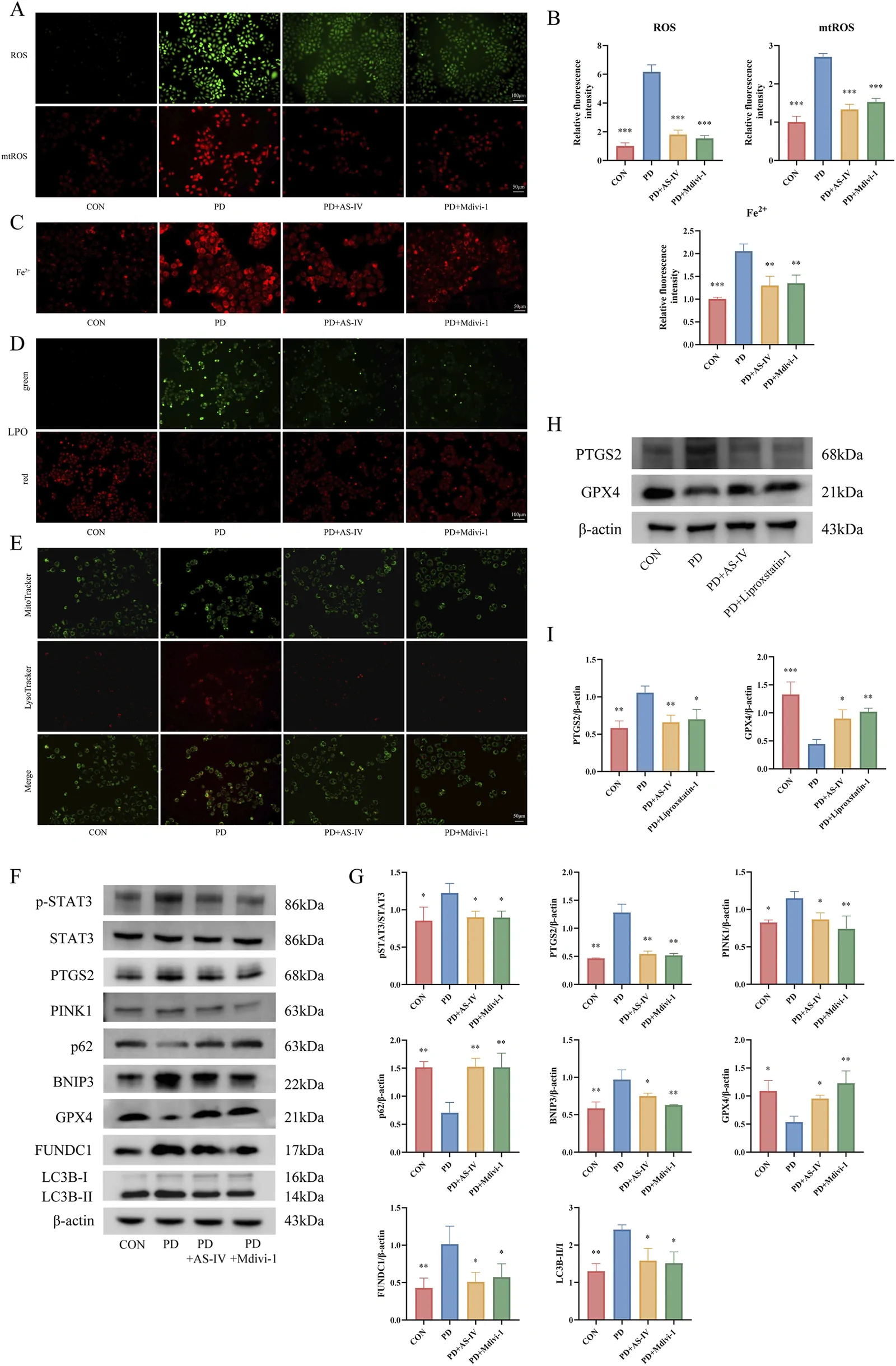
AS-IV inhibits PDF-induced mitophagy to attenuate ferroptosis in PMCs. HMrSV5 cells were treated with 3% dextrose PDF for 24 h in the absence or presence of AS-IV (200 μM), Mdivi-1 (10 μM), or Liproxstatin-1 (200 nM). Groups for panels A-G: Control, PD, PD + AS-IV, PD + Mdivi-1. Groups for panels H-I: Control, PD, PD + AS-IV, PD + Liproxstatin-1. **(A–E)** Representative fluorescence images showing intracellular ROS (DCFH-DA), mitochondrial ROS (MitoSOX Deep Red), Fe^2+^ (FerroOrange), LPO (C11-BODIPY 581/591), and mitochondria-lysosome colocalization (MitoTracker Green/LysoTracker Red) in HMrSV5 cells. Scale bars, 50 μm (mtROS, colocalization, and Fe^2+^) and 100 μm (ROS and LPO). Quantitative analysis of ROS, mtROS, and Fe^2+^ levels is shown (n = 3). **(F,G)** Expression of p-STAT3, STAT3, PTGS2, PINK1, p62, BNIP3, GPX4, FUNDC1, and LC3B in HMrSV5 cells was analyzed by Western blotting. Band intensities of PTGS2, PINK1, p62, BNIP3, GPX4, and FUNDC1 were normalized to β-actin; LC3B was quantified as the LC3B-II/LC3B-I ratio; and p-STAT3 was quantified as the p-STAT3/STAT3 ratio (n = 3). **(H,I)** Expression of PTGS2 and GPX4 in HMrSV5 cells was analyzed by Western blotting, and band intensities were normalized to β-actin (n = 3). Data are presented as mean ± SD. One-way ANOVA with Tukey’s multiple-comparisons test was used. *p < 0.05, **p < 0.01, ***p < 0.001 vs. PD.

AS-IV was administered to PDF-exposed cells to further investigate the underlying mechanism of AS-IV’s peritoneal protective effects. Fluorescence staining showed that AS-IV partially reversed the PDF-induced increase in ROS and mtROS levels, exerting antioxidant effects (Figures 5A,B). Additionally, AS-IV reduced mitochondria-lysosome colocalization, downregulated mitophagy-related proteins (PINK1, BNIP3, FUNDC1, and the LC3B-II/I ratio), and upregulated p62 expression (Figures 5E–G). Fluorescence staining and WB analysis further showed that AS-IV treatment decreased Fe^2+^ and LPO levels (Figures 5B–D) while upregulating GPX4 expression and downregulating PTGS2 expression (Figures 5F,G). Similarly, WB analysis showed that the ferroptosis inhibitor Liproxstatin-1 reversed the PDF-induced changes in GPX4 and PTGS2 expression (Figures 5H,I). Furthermore, siRNA-mediated knockdown of PINK1 were used in PMCs. WB analysis showed that siPINK1 significantly attenuated PD-induced ferroptosis, and enhanced the protective effect of AS-IV against ferroptosis (Supplementary Information B). These findings indicate that AS-IV attenuated ferroptosis by inhibiting mitophagy.

Moreover, it has been reported in the literature that STAT3 is a key target of glucose-induced peritoneal injury (Fu et al., 2022; Li N. et al., 2024). WB analysis showed that PDF exposure promoted STAT3 phosphorylation, which was reduced following AS-IV and Mdivi-1 treatment (Figures 5F,G). These findings suggest that AS-IV may attenuate PDF-induced ferroptosis in PMCs by inhibiting mitophagy and suppressing STAT3 phosphorylation.

### 3.6 AS-IV attenuates mitophagy to alleviate ferroptosis in PMCs by suppressing STAT3 phosphorylation

**FIGURE 6:**
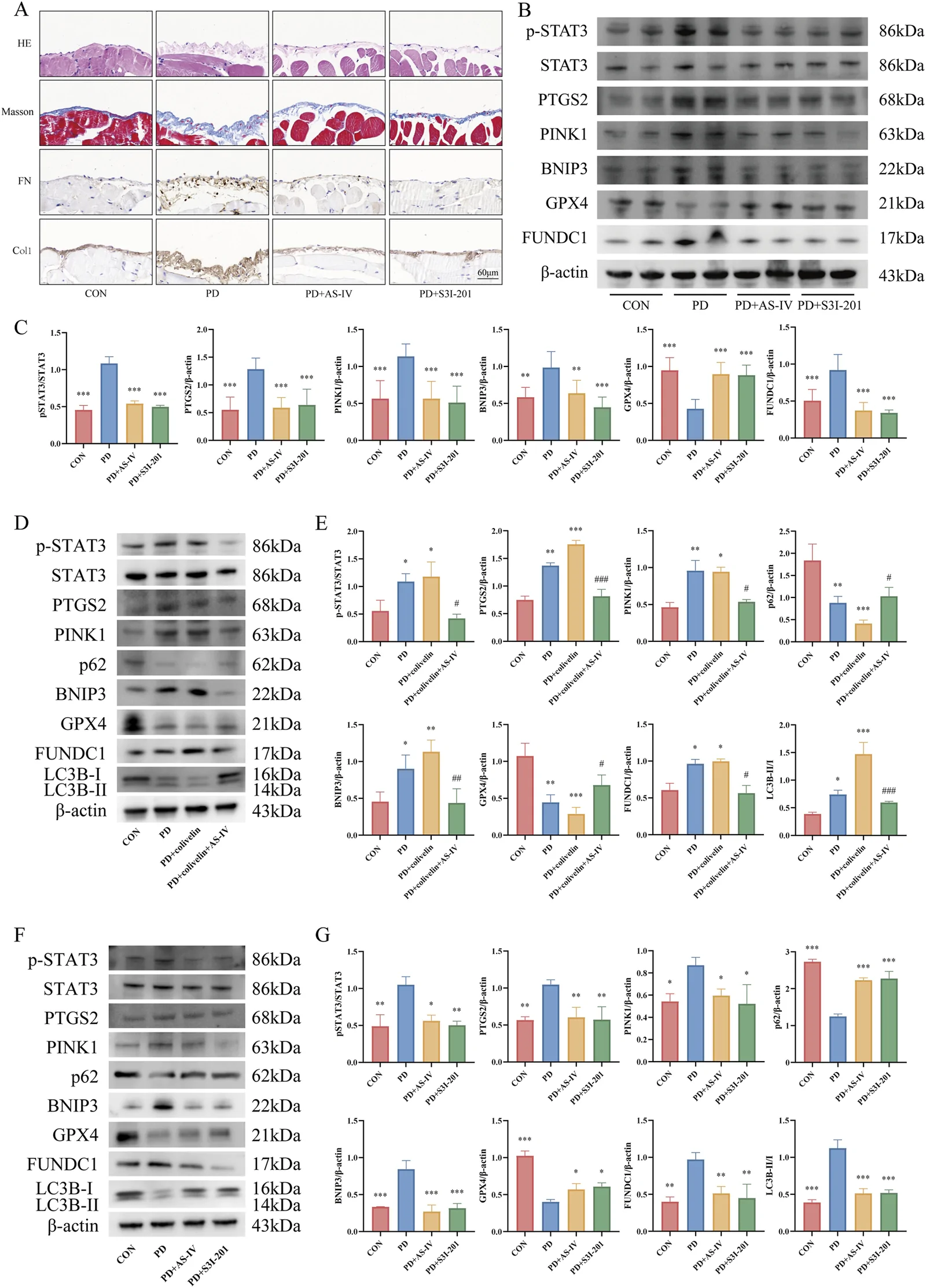
AS-IV inhibits mitophagy to attenuate ferroptosis in PMCs by inhibiting STAT3 phosphorylation. For *in vivo* experiments **(A–C)** mice received daily intraperitoneal injection for 4 weeks with saline (Control), 4.25% dextrose PDF (100 mL/kg, PD), PD + AS-IV (15 mg/kg), or PD + S3I-201 (10 mg/kg). Tissues were collected at the end of week 4. For *in vitro* experiments, HMrSV5 cells were treated with 3% dextrose PDF for 24 h in the absence or presence of AS-IV (200 μM), colivelin (1 μM), or S3I-201 (100 μM). Groups for **(D,E)** Control, PD, PD + colivelin, PD + colivelin + AS-IV. Groups for panels **(F,G)**: Control, PD, PD + AS-IV, PD + S3I-201. **(A)** Representative HE staining, Masson staining, and immunohistochemical staining of peritoneal tissues. Scale bar, 60 μm. **(B,C)** Expression of p-STAT3, STAT3, PTGS2, PINK1, BNIP3, GPX4, and FUNDC1 in mouse peritoneal tissues was analyzed by Western blotting. Band intensities of PTGS2, PINK1, BNIP3, GPX4, and FUNDC1 were normalized to β-actin, and p-STAT3 was quantified as the p-STAT3/STAT3 ratio (n = 4). **(D,E)** Expression of p-STAT3, STAT3, PTGS2, PINK1, p62, BNIP3, GPX4, FUNDC1, and LC3B in HMrSV5 cells (Control, PD, PD + colivelin, PD + colivelin + AS-IV) was analyzed by Western blotting, with quantification as described above (n = 3). **(F,G)** Expression of p-STAT3, STAT3, PTGS2, PINK1, p62, BNIP3, GPX4, FUNDC1, and LC3B in HMrSV5 cells (Control, PD, PD + AS-IV, PD + S3I-201) was analyzed by Western blotting, with quantification as described above (n = 3). Data are presented as mean ± SD. One-way ANOVA with Tukey’s multiple-comparisons test was used. For **(C**,**G)** *p < 0.05, **p < 0.01, ***p < 0.001 vs. PD. For **(E)** *p < 0.05, **p < 0.01, ***p < 0.001 vs. Control; ^#^ *p* < 0.05, ^##^ *p* < 0.01, ^###^ *p* < 0.001 vs. PD + colivelin.

Molecular docking analysis suggests a potential interaction between AS-IV and STAT3 (Supplementary Information C). The STAT3 phosphorylation inhibitor S3I-201 and the agonist colivelin were used to investigate the role of STAT3 activation on mitophagy and ferroptosis during PD and the therapeutic effects of AS-IV (Urban et al., 2022; Song Q. H. et al., 2024). *In vivo*, peritoneal tissue from the PD group exhibited significant thickening, irregular arrangement and partial detachment of PMCs, submesothelial collagen deposition, and extensive inflammatory infiltration. Additionally, collagen 1 and FN expression were upregulated, suggesting structural alterations (Figure 6A).

Conversely, treatment with S3I-201 led to reduced peritoneal thickness, collagen deposition, and inflammatory infiltration while downregulating collagen 1 and FN expression. These effects were similar to those observed following intraperitoneal AS-IV injection (Figure 6A). Furthermore, GPX4 expression decreased, and PTGS2 expression increased in the PD group; these changes were reversed by both S3I-201 and AS-IV treatment (Figures 6B,C), indicating that inhibiting STAT3 phosphorylation mitigates ferroptosis caused by PD.

**FIGURE 7:**
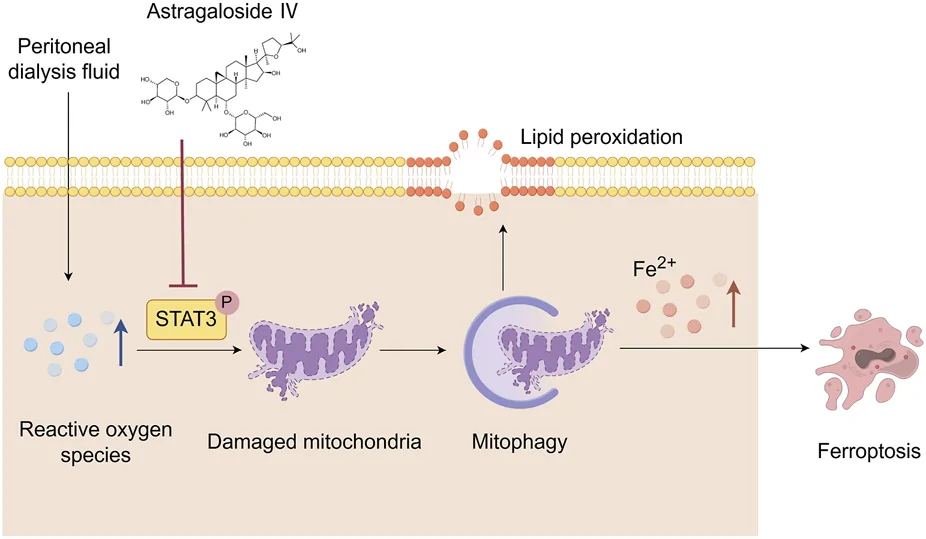
Schematic illustration of the proposed mechanism. PD fluid induces ROS overproduction in PMCs, resulting in mitochondrial damage and excessive mitophagy, which drives ferroptosis. AS-IV suppresses STAT3 activation, thereby alleviating mitochondrial damage and restraining mitophagy, ultimately protecting PMCs against ferroptosis.

*In vitro*, PD treatment resulted in increased levels of mitophagy-related proteins (PINK1, BNIP3, FUNDC1, and the LC3B-II/I ratio), decreased p62 and GPX4 expression, increased PTGS2 expression, and enhanced STAT3 phosphorylation. These changes were exacerbated by colivelin exposure, but reversed by AS-IV treatment (Figures 6D,E). The effect of AS-IV is similar to S3I-201 (Figures 6F,G). Collectively, these findings suggest that AS-IV may protect PMCs by attenuating mitophagy and ferroptosis through the suppression of STAT3 activation (Figure 7).

## 4 Discussion

PD-induced injury to PMCs is a major barrier to long-term treatment efficacy, making it essential to clarify the underlying mechanisms and identify protective strategies (Ito et al., 2024). In the present study, we found that oxidative stress is an early driver of mitophagy and ferroptosis in PMCs, as supported by the protective effects of NAC, a ROS scavenger. Consistent with these observations, mitophagy and ferroptosis markers were elevated during PD, and modulation of mitophagy with agonists and inhibitors, together with ferroptosis inducers, further confirmed that mitophagy contributes to ferroptotic regulation. On this basis, our data indicated that STAT3 is activated during PD and serves as an important upstream regulator of mitophagy-dependent ferroptosis. Finally, both *in vivo* and *in vitro* analyses demonstrated that AS-IV inhibits STAT3 activation and protects PMCs by suppressing this mitophagy-dependent ferroptosis axis.

Oxidative stress plays a key role in peritoneal injury pathogenesis, primarily due to the non-biocompatible components of PDF. Clinical studies have reported that PD patients exhibited elevated MDA, reduced antioxidant enzyme activity, and frequent vitamin D insufficiency, collectively supporting a redox-imbalanced state (Shah et al., 2005; Zhang et al., 2021). In this context, vitamin D is relevant not only as an isolated variable, but also as a potential modifier of oxidative stress (Sosa-Díaz et al., 2022). As key regulators of oxidative stress, ROS are essential for maintaining cellular redox homeostasis through their production and scavenging mechanisms (Sies et al., 2022). In this study, PDF exposure increased ROS and mtROS production in PMCs, elevated peritoneal ROS levels, and increased serum MDA levels in mice. NAC attenuated these changes, supporting the involvement of oxidative stress. In parallel, AS-IV exhibited antioxidant effects and mitigated peritoneal injury.

Oxidative stress regulates STAT3 not only by enhancing its phosphorylation but, in some contexts, also by increasing its expression. In human breast cancer cells, H_2_O_2_, a representative ROS, was shown to increase STAT3 protein expression and induced mitochondria importation (Wang et al., 2018). Similarly, both STAT3 mRNA and protein levels were elevated in H_2_O_2_-treated spinal cord neurons (Qiu et al., 2021). However, other studies have reported that under oxidative stimulation, STAT3 Tyr705 phosphorylation increased without a significant change in total STAT3 expression, and that ROS scavengers could block STAT3 activation (Butturini et al., 2020; Tao et al., 2021). STAT3 signaling has been implicated in oxidative stress regulation across various diseases (Li Q. et al., 2024; Yang et al., 2024), including peritoneal fibrosis, where asiaticoside modulated JAK/STAT3 signaling through its antioxidant effects (Sun et al., 2023). In acute ischemic stroke, AS-IV has been reported to reduce STAT3 phosphorylation at Tyr705, inhibiting CCL2-mediated brain infiltration and activation of NK cells (Li S. C. et al., 2022). In the present study, molecular docking analysis supports the possibility of a potential interaction between AS-IV and STAT3. Although the predicted binding site was distinct from the phosphorylation site examined in our experiments, this does not necessarily indicate a contradiction, as ligand binding at a non-phosphorylation region may still modulate STAT3 activation through allosteric effect, including altered phosphatase accessibility (Changeux, 2012; Yu et al., 2025).

Mitophagy, which removes dysfunctional mitochondria, is essential for maintaining intracellular homeostasis (Lu et al., 2023). Emerging evidence links STAT3 activation to mitophagy-related processes. In endotoxemia-induced cardiomyopathy, STAT3 was shown to regulate mitophagy by binding to response elements in the *Pink1* promoter and promoting *Pink1* transcription (Song Q. X. et al., 2024). In addition, STAT3 has been reported to function as a transcription factor for *FUNDC1*, binding to its promoter to regulate FUNDC1-dependent mitophagy in cardiomyocytes (Jiang et al., 2022). These findings accord with our observations, which showed that STAT3 signaling and mitophagy were both activated in MGO- and PDF-induced peritoneal oxidative stress models, whereas treatment with either S3I-201 or AS-IV reversed these changes, attenuated ferroptosis in PMCs, and ameliorated peritoneal injury.

Ferroptosis, an oxidation-related form of cell death, has not been previously reported in PD-related peritoneal fibrosis. Excessive ROS production and lipid peroxidation contribute to damage of the plasma membrane’s lipid bilayer, triggering ferroptosis (Jiang et al., 2021). Recent studies have found that mitochondrial dysfunction exacerbates lipid peroxidation, increases mtROS production, reduces mitochondrial membrane potential, and disrupts mitochondrial oxidative stress regulation, thereby preventing ferroptosis suppression (Chen G. H. et al., 2022). In damaged astrocytes, NADPH oxidase 4 overexpression enhanced mtROS production and lipid peroxidation, leading to ferroptosis (Park et al., 2021). GPX4 suppresses ferroptosis by decreasing peroxidized membrane lipid and terminating the complex lipid peroxidation cascade (Imai et al., 2017; Maiorino et al., 2018). In diabetic nephropathy, inhibition of GPX4 ubiquitination suppressed renal oxidative stress injury, thereby preventing ferroptosis (Chen J. et al., 2022). Consistent with the literature, our data demonstrated that PDF induced ferroptosis both *in vivo* and *in vitro*, as evidenced by elevated Fe^2+^ and LPO levels and altered expression of ferroptosis markers; these changes were effectively reversed by either Liproxstatin-1 or AS-IV.

The interplay between mitophagy and ferroptosis has been reported in several diseases, including Alzheimer’s disease, liver injury, and neurotoxicity studies (Li J. H. et al., 2022; Qian et al., 2022; Bi et al., 2024). Ferroptosis has been identified as a mitophagy-dependent form of cell death, and mitophagy inhibition has been shown to protect cells from ferroptosis. Suppression of O-GlcNAcylation promotes ferritinophagy, leading to the accumulation of labile iron in mitochondria, which contributes to mitochondrial fission and subsequent mitophagy. Mitophagy may further release mitochondrial iron pools. Together, ferritinophagy and mitophagy enhance lipid peroxidation and promote ferroptosis (Yu et al., 2022). The pharmacological compound WJ460, which targets myoferlin, has been shown to trigger mitophagy and ROS accumulation, ultimately leading to lipid peroxidation and ferroptosis. These effects can be mitigated by Mdivi-1 or iron chelators, which provide cellular protection (Rademaker et al., 2022). These findings are consistent with our data, which showed that CCCP induced both mitophagy and ferroptosis, whereas Mdivi-1 attenuated these effects. Additionally, siPINK1 attenuated PDF-induced ferroptosis and further strengthened the anti-ferroptotic effect of AS-IV.

However, this study has several limitations. First, we did not investigate the precise molecular mechanisms by which STAT3 regulates mitophagy and mitophagy regulates ferroptosis. Second, this was a mechanistic exploratory study using a single AS-IV dose; therefore, the concentration used in the study should be interpreted as a mechanistic experimental condition. Although dissolving AS-IV in PDF may be a promising therapeutic strategy, published studies indicate relatively rapid *in vivo* clearance of AS-IV after parenteral administration, suggesting that fixed 24-h *in vitro* exposure may not fully reflect *in vivo* exposure kinetics (Zhang et al., 2006; Xu et al., 2013). Future studies should elucidate the STAT3-mitophagy-ferroptosis signaling axis in greater detail, include multi-dose experiments, and incorporate clinically feasible delivery strategies to strengthen translational relevance.

In conclusion, this study supported a model in which oxidative stress-associated excessive mitophagy promotes ferroptosis in PMCs during PD and identified AS-IV as an effective modulator of this axis by suppressing STAT3 activation. CCCP-induced mitophagy increased Fe^2+^ and LPO levels, whereas Mdivi-1 attenuated erastin-induced ferroptosis, indicating that mitophagy contributes to ferroptosis. On this basis, AS-IV suppressed STAT3 activation, restrained mitophagy, and thereby attenuated ferroptosis in PMCs. Given the single-dose design, these findings should be interpreted with caution and warrant further validation in dose-ranging studies.

## Data Availability

The original contributions presented in the study are included in the article/Supplementary Material, further inquiries can be directed to the corresponding authors.

## Supplementary material

SUPPLEMENTARY INFORMATION Additional validation experiments. **(A)** CCK-8 assay of HMrSV5 cell viability after AS-IV treatment at different concentrations and after 200 μM AS-IV treatment for different durations. Data are presented as mean ± SD. Statistical analysis was performed using one-way ANOVA followed by Tukey’s multiple-comparisons test, n = 3, **p* < 0.05. **(B)** Representative Western blot bands showing the effects of PINK1 knockdown on PD-induced PTGS2 and GPX4 protein expression in PMCs, and the corresponding modulation by AS-IV. **(C)** Molecular docking analysis of AS-IV with STAT3. Docking visualization was generated using PyMOL 3.0, with a predicted binding energy of −7.961 kcal/mol.
